# Registration of Aerial Optical Images with LiDAR Data Using the Closest Point Principle and Collinearity Equations

**DOI:** 10.3390/s18061770

**Published:** 2018-06-01

**Authors:** Rongyong Huang, Shunyi Zheng, Kun Hu

**Affiliations:** 1 Guangxi Laboratory on the Study of Coral Reefs in the South China Sea, Guangxi University, Nanning 530004, China; 2 School of Marine Sciences, Guangxi University, Nanning 530004, China; 3 Coral Reef Research Centre of China, Guangxi University, Nanning 530004, China; 4 School of Remote Sensing and Information Engineering, Wuhan University, Wuhan 430079, China; syzheng@whu.edu.cn; 5 Collaborative Innovation Center for Geospatial Technology, Wuhan University, Wuhan 430079, China; 6 Institute of Electronics, Chinese Academy of Sciences, Beijing 100190, China; hu.1775@osu.edu; 7 Key Laboratory of Technology in Geo-Spatial Information Processing and Application System, Chinese Academy of Sciences, Beijing 100190, China; 8 Department of Civil, Environmental and Geodetic Engineering, Ohio State University, Columbus, OH 43210, USA

**Keywords:** registration, aerial Image, LiDAR, point cloud, collinearity equation

## Abstract

Registration of large-scale optical images with airborne LiDAR data is the basis of the integration of photogrammetry and LiDAR. However, geometric misalignments still exist between some aerial optical images and airborne LiDAR point clouds. To eliminate such misalignments, we extended a method for registering close-range optical images with terrestrial LiDAR data to a variety of large-scale aerial optical images and airborne LiDAR data. The fundamental principle is to minimize the distances from the photogrammetric matching points to the terrestrial LiDAR data surface. Except for the satisfactory efficiency of about 79 s per 6732 × 8984 image, the experimental results also show that the unit weighted root mean square (RMS) of the image points is able to reach a sub-pixel level (0.45 to 0.62 pixel), and the actual horizontal and vertical accuracy can be greatly improved to a high level of 1/4–1/2 (0.17–0.27 m) and 1/8–1/4 (0.10–0.15 m) of the average LiDAR point distance respectively. Finally, the method is proved to be more accurate, feasible, efficient, and practical in variety of large-scale aerial optical image and LiDAR data.

## 1. Introduction

Light detection and ranging (LiDAR) has been an indispensable technology in the field of surveying and mapping, and many researchers agree that photogrammetry and LiDAR are fairly complementary for more accurate and complete products and a higher automation level of processes [1,2,3,4,5]. As a result, photogrammetry and LiDAR were integrated for plenty of applications, e.g., tree detection [6,7], building detection [8], change detection [9], true orthophoto generation [10], 3D city model creation [11,12,13,14,15], and landscape roughness estimation [16], etc.

As is known, integration of photogrammetry and LiDAR can only be implemented after the geometric registration, making the photogrammetric model and the LiDAR data relative to a common reference frame [17,18,19]. Currently, direct georeferencing (DG) of airborne sensors with GPS/INS is a widely accepted approach in the airborne mapping industry [20,21,22]. Hence, the geometric registration of aerial images with LiDAR data may be achieved by integrating the aerial camera and the laser scanner device with GPS/INS directly.

However, until now, the geometric misalignments between the optical images and the LiDAR data are still difficult to avoid by using DG in actual projects [3,20,21]. Possible reasons are as follows: (1) the position and orientation records of some early images were lost due to human factors; (2) as collected by different platforms or in different periods, some optical images and LiDAR data may be referenced to different national coordinate frames without known coordinate transformation parameters; (3) there are some system errors in actual integrated sensor orientation (ISO) system, such as lever-arms, boresights, synchronizations, and interior orientation parameters, etc. Therefore, it is still necessary to research on the geometric registration of aerial optical images with LiDAR data, and this is what we focus on in this paper.

Geometric registration of aerial images with LiDAR data can be considered to orient 2D images to 3D point clouds, so the registration can be achieved by using photogrammetric orientation procedures. In such procedures, the control points are collected from the LiDAR point clouds. One common way to collect control points for the registration is to select conjugating pairs interactively. Wu et al. [23] presented a two-step displacement correction algorithm for registration of aerial images and LiDAR data with interactive point pairs selecting. Kurz et al. [24] registered spectral panoramic imagery and LiDAR data with control point measurement and block adjustment. Some others [25,26,27] registered the aerial images with LiDAR data relying on manual vanishing point detection. The problem is that it is hard to pick out enough evenly distributed conjugate pairs even with significant manual efforts, and errors from the selected pairs will be propagated and enlarged by the transformation models [28,29].

Another way to collect control points is to automatically extract and match salient feature points between the optical images and the LiDAR intensity images. For example, Toth and Ju [30] matched the features of the optical satellite images to LiDAR intensity images for their georeferencing; Wang et al. [27] presented a study on a multisource image automatic registration system (MIARS) based on the scale-invariant feature transform (SIFT).

Since the LiDAR intensity images would provide better similarity to the optical images than the elevation values within a digital surface model (DSM), incorporating LiDAR intensity images into the registration of 2D image with 3D LiDAR data should have great potential to enhance the registration accuracy [2]. Nevertheless, matching between image pixels and LiDAR intensities are not always available because of the irregular and discrete distribution of the LiDAR data, the low density of the point clouds, and the nonlinear relationship between LiDAR intensity and the aerial optical images, etc.

Some other artificial point features, such as roof centroids [31,32] and building corners [33,34] are also used as the control points to register airborne LiDAR data with aerial optical images. They are feasible in urban areas, but will become difficult in rural areas. The reason is that there are few artificial feature point in rural areas.

Point cloud-based registration is also applied to the registration of aerial optical images with LiDAR data. In such approaches, the optical images are transformed into 3D point clouds by using photogrammetric image matching or structure from motion (SFM), and the registration is then implemented by 3D motion analysis methods. Habib et al. [35] generated the 3D straight-line pairs from the optical images and the LiDAR data respectively. Accordingly, the transformation between the photogrammetric coordinate system and the LiDAR reference frame is then established by using those 3D straight-line pairs. Kim et al. [36] presented a registration method based on plane-feature correspondence between the LiDAR depth map and the photogrammetric depth map generated from the optical images. Zhao et al. [37] used stereo vision techniques to infer 3D structure from video sequences followed by the 3D to 3D registration with an iterative closest-point (ICP) algorithm [38]. Pothou et al. [39] proposed an algorithm for the registration of the optical images and the LiDAR data, based on the minimization of the distances between the points of one surface-to-surface patch of the other surface. Teo and Huang [40] also developed similar methods to align 2D images and 3D LiDAR data to a common reference frame using improved ICP algorithm. Point cloud-based methods are very simple and convenient, but require much more accurate 3D multi-view reconstruction. In other words, to eliminate the deformation of the point clouds generated from the optical images, camera calibration must be done before collecting the optical images, or plenty of ground control points must be set up for a self-calibration bundle adjustment. Otherwise, 3D to 3D point cloud-based registration can only apply to registration on small blocks.

Linear and planar feature-based registration is another important registration approach. Such an approach refers to orienting the optical images to the LiDAR data via matching linear or planar features. Schenk and Csathó [4] proposed the sensor-invariant feature concept, such as linear features and planar features, for the registration of the aerial optical images and the LiDAR data in 2002. Habib et al. [19,41] incorporated the straight-line features derived from the LiDAR data and the optical images to a photogrammetric triangulation based on a modified coplanar constraint. Shorter and Kasparis [42] developed a registration method based on building roofs resent in both the optical images and the LiDAR data. In the method, binary masks of the buildings extracted from the LiDAR data and the aerial optical images were used to register the two data sets. Deng et al. [43] presented a registration algorithm by matching the straight-line pairs detected from the LiDAR data and the optical images by using generalized point photogrammetry. Ding et al. [44] made use of the vanishing points to extract some features named 2DOCs (2D orthogonal corners) for refining the camera parameters refer to the point clouds. Wang and Neumann [45] further proposed a novel feature called 3CS (3 connected segments) to develop a robust automatic registration approach, which are claimed more distinctive than 2DOCs. Choi et al. [46] also proposed a method to simultaneously register optical images with LiDAR data, using some area and linear primitives as the ground control features. Yang and Chen [47] proposed a novel automatic registration method for mini-UAV sequent images and LiDAR data: coarse registration are generated by extracting some building outlines and corners and making use of SFM and multi-view stereo (MVS) algorithms, then an ICP algorithm is further employed to refine the registration. Safdarinezhad et al. [48] utilized shadow contours to align QuickBird sub-images with aerial LiDAR data, and obtained the RMSEs of 0.85–1.30 pixels. Javanmardi et al. [49] presented a road feature-based framework for automatic georeferencing of mobile mapping system point cloud with the aerial images for urban areas. As the accuracy appears to be much lower while using natural features than artificial features [3], linear and planar feature-based registration is mainly suitable for areas that are rich in artificial linear and planar features, such as buildings, roads, and urban areas, etc.

Finally, mutual information-based registration methods have been proposed in the field of medical imaging over the past few decades [50], and its promising results have encouraged the computer vision and remote sensing communities to exploit the approach for multi-sensor data registration [51,52]. Mastin et al. [53] exploited the statistical dependency in urban scenes of the optical appearance with the LiDAR data, and proposed an application of mutual information (MI) registration methods. Parmehr et al. [54,55,56] also researched on a combined mutual information (CMI) technique for the registration of the optical imageries and LiDAR data several years. They have produced a similarity measure that can exploit the inherently registered LiDAR intensity and point cloud data to improve the robustness of the registration. Mutual information-based registration methods are usually calculated by using derivative-free optimization (DFO) [53,54,56], such as Powell’s optimization or downhill simplex optimization. As a result, mutual information-based methods still need much further improvements to reduce the computational burden, or they will be difficult to apply to large-scale aerial images and LiDAR data in practice.

On the basis of the collinearity equation model, Zheng et al. [57] have proposed a flexible and convenient method for the registration of optical images with terrestrial LiDAR data implemented by minimizing the distances from the photogrammetric matching points to the terrestrial LiDAR data surface. The method can greatly reduce the manual work and errors, and obtain a relative high accuracy. What is more, the method has the following advantages: (1) the registration can be implemented without linear and planar feature extraction and segmentation; (2) the non-rigid deformation caused by lens distortion can be effectively eliminated with the introduction of lens distortion parameters; (3) neither camera calibration nor extra control points for a self-calibration are required. However, as the method was designed mainly for terrestrial data rather than airborne data, the method was not further tested on aerial images and LiDAR point clouds.

In this paper, we extend the method proposed by Zheng et al. [57] from close-range images and terrestrial point clouds to large-scale aerial optical images and airborne LiDAR data. As the calculation can follow some derivative-based optimization methods, we do not worry about the computational burden for the extension. On the other hand, the extension can keep all the advantages of the method proposed by Zheng et al. [57]. In other word, in addition to the extension to large-scale airborne data, the method can inherit the advantages as follows: (1) the method can be implemented without linear or planar feature extraction and segmentation in the LiDAR data; (2) if necessary, the method can also eliminate the non-rigid deformation caused by lens distortion without extra camera calibration or a large number of ground control points, i.e., neither camera calibration nor plenty of control points are necessary for the method. Hence, in contrast to some linear and planer feature-based methods, the extended method is able to adapt to both urban and rural areas.

## 2. Materials and Methods

### 2.1. Materials

Four different data sets are used to test the extended method on the registration of aerial optical images and airborne LiDAR data. Data I and II are located in Xinjiang and Guangdong, China, respectively. Data III and IV are both located in Guangxi, China. Both the images and the point clouds of data I were captured in 2014, while those of data II were obtained in 2011. Data III and IV share the same LiDAR data, which was acquired in 2010. But their optical images were captured in 2010 and 2012 respectively. The optical images of data II were captured by a Rollei Metric AIC Pro digital camera, and the LiDAR data was acquired by a Harrier 68i laser scanning device (Trimble, Sony Weil, CA, USA). The horizontal and vertical accuracy of the laser scanning device approximate 0.25 m and 0.15 m respectively. The camera and laser scanning device versions of other data sets are unknown. More information on these data sets is presented in Table 1.

### 2.2. Fundamental Geometric Relationship

**Figure 1:**
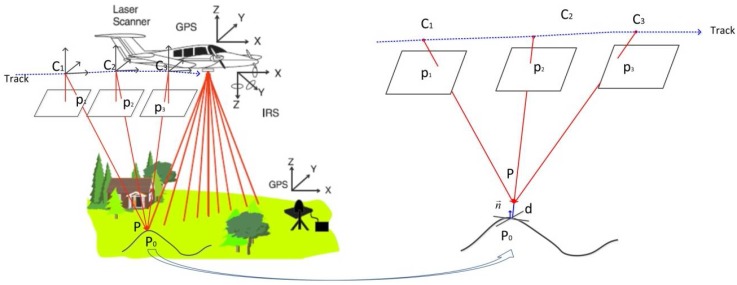
Fundamental geometric relationship between aerial optical images and LiDAR data.

With the registration of aerial optical images with LiDAR data, we refer to the process of establishing a common reference frame for the two data types. Thus, the fundamental geometric relationship between the images and the point clouds must be clearly illustrated first. The purpose is to find useful redundancy for the registration. The fundamental geometric relationship is shown in Figure 1, using a typical airborne LiDAR system as an example.

Photogrammetry is a traditional method of reconstructing surfaces, in which a 3D point or linear feature can be reconstructed from two or more overlapping aerial images [4]. If the interior and exterior orientation parameters of the images have been known, the 3D points can be intersected by using conjugate rays, as shown in Figure 1. It means that the crucial step of photogrammetry is the image matching to identify the same features from different images. Conversely, interior and exterior orientation parameters can also be exactly refined by using plenty of conjugate image points, e.g., bundle adjustment is such a tool to jointly refine the optimal 3D structure and the interior and exterior orientation parameters [58].

LiDAR has been proven to be a promising system that can sample the 3D points of the reflective surface effectively and accurately [4]. As shown in Figure 1, the laser points are computed from the navigation data (GPS/INS) and range measurements, so there is no inherent redundancy in the computation of a laser point. However, it is important to realize that the surfaces captured by photogrammetry and LiDAR are actually the same, and this produces the redundancy to connect the aerial optical images and the airborne LiDAR data, as shown in the right of Figure 1.

### 2.3. Parameterization of the Geometric Relationship

Based on the fundamental geometric relationship, suppose P is a 3D ground point that can be intersected by the conjugate rays of several different optical images, as shown in Figure 1. Then the geometry of the optical images is parameterized by collinearity equations as follows [59]:

(1) $$\{\begin{array}{c} x-x_{0}-\Delta x=-f\frac{a_{1}(X-X_{s})+b_{1}(Y-Y_{s})+c_{1}(Z-Z_{s})}{a_{3}(X-X_{s})+b_{3}(Y-Y_{s})+c_{3}(Z-Z_{s})}\\ y-y_{0}-\Delta y=-f\frac{a_{2}(X-X_{s})+b_{2}(Y-Y_{s})+c_{2}(Z-Z_{s})}{a_{3}(X-X_{s})+b_{3}(Y-Y_{s})+c_{3}(Z-Z_{s})} \end{array},$$

where:

(2) $$\left\{ \begin{array}{l} \Delta x=(x-x_{0})(k_{1}r^{2}+k_{2}r^{4})+p_{1}[r^{2}+2{\left(x-x_{0}\right)}^{2}]+2p_{2}(x-x_{0})(y-y_{0})\\ \Delta y=(y-y_{0})(k_{1}r^{2}+k_{2}r^{4})+p_{2}[r^{2}+2{\left(y-y_{0}\right)}^{2}]+2p_{1}(x-x_{0})(y-y_{0})\\ r=\sqrt{{\left(x-x_{0}\right)}^{2}+{\left(y-y_{0}\right)}^{2}} \end{array}\right..$$

In addition, *x* and *y* are the coordinates of the image point, $x_{0}$ and $y_{0}$ are the coordinates of the principle point, f is the principle distance, $k_{1}$, $k_{2}$, $p_{1}$ and $p_{2}$ are the lens distortion parameters, X, Y and Z are the object space coordinates of the ground point P, $X_{s}$, $Y_{s}$ and $Z_{s}$ are the object space coordinates of the perspective center, and $a_{i}$, $b_{i}$ and $c_{i}$ (i=1,2,3) are the nine elements of the rotation matrix formed by three rotation angles φ, ω and κ.

The collinearity equations contain three categories of unknowns: exterior orientation parameters, object point coordinates, and intrinsic parameters (the principle point coordinates, principle distance, and lens distortion parameters). Similar to self-calibration, the corresponding error equations are illustrated by using the first-order Taylor series expansion as follows:

(3) $$V_{x,y}=A_{x,y}\Delta t+B_{x,y}\Delta p+C_{x,y}\Delta i-L_{x,y},$$

where $V_{x,y}={\left(\begin{array}{cc} v_{x} & v_{y} \end{array}\right)}^{T}$ is the residual vector of image point observations ${\left(\begin{array}{cc} x & y \end{array}\right)}^{T}$, and Δt, Δp, and Δi stand for the correction terms of the exterior orientation parameters, the object point coordinates, and the intrinsic parameters respectively. $A_{x,y}$, $B_{x,y}$ and $C_{x,y}$ refer to the corresponding first-order partial derivatives of the error equations, $L_{x,y}$ the constant item calculated by using the approximate values of the unknowns.

Furthermore, since the surface generated by the images is actually the same as the one captured by LiDAR, the closest point principle proposed by Zheng et al. [57] can also be used in this paper to connect the aerial images and the LiDAR data. Although the coordinates of the 3D ground point P are seen as unknowns and we cannot find the exact correspondence from the LiDAR data, P should be located on the LiDAR data surface. In other words, whereas there are unpredictable random errors in both the image matching and the LiDAR point clouds, P should be forced to be as close to the LiDAR data surface as possible.

Accordingly, suppose the distance from P to the LiDAR data surface is d, and d should be possibly and infinitely close to zero, as shown in the right of Figure 1. Suppose further that the real surface is smooth enough to be fitted locally by the tangent plane, and the coordinates of the closest point of P in the LiDAR data and the corresponding normal vector are $P_{0}={\left(\begin{array}{ccc} X_{0} & Y_{0} & Z_{0} \end{array}\right)}^{T}$ and $\vec{n}={\left(\begin{array}{ccc} n_{x} & n_{y} & n_{x} \end{array}\right)}^{T}$, respectively, then d can be estimated by using the distance from P to the tangent plane at $P_{0}$ as follows:

(4) $$d\approx {\vec{n}}^{T}(P–P_{0})\approx 0.$$

The error equation corresponding to equation (4) is also formed by first-order Taylor series expansion:

(5) $$v_{p}={\vec{n}}^{T}\Delta p–{\vec{n}}^{T}(P_{0}–P^{\left(0\right)}),$$

where $P^{\left(0\right)}$ is the initial value vector of the coordinates of P. It should be noted that the normal vector $\vec{n}$ at $P_{0}$ can be estimated by fitting a local plane with several approximate points around $P_{0}$, so $\vec{n}$ is regarded as known.

### 2.4. Bundle Adjustment Model and Solution

The exterior orientation parameters, the object point coordinates, and the intrinsic parameters should satisfy Equations (3) and (5) at the same time. Therefore, to further consider the virtual observation equations of the intrinsic parameters, the error equations can be written as follows:

(6) $$\left\{ \begin{array}{ccc} V_{}= & A\Delta T+B\Delta P+C\Delta I-L_{} & W\\ V_{P}= & \;N\Delta P\;-L_{P} & W_{P}\\ V_{I}= & \;\Delta I-L_{I} & \;W_{I} \end{array}\right.,$$

where V is the residual vector consisting of $V_{x,y}$ in Equation (3), $V_{P}$ is the residual vector consisting of $v_{p}$ in Equation (5), and $V_{I}$ is the error vectors of the virtual observation equations of the intrinsic parameters; A, B, and C are the partial derivative matrixes, which consists of all $A_{x,y}$, $B_{x,y}$, and $C_{x,y}$; ΔT, ΔP, and ΔI are the corresponding correction terms, which consists of all Δt, Δp, and Δi in Equation (3); N is the coefficient matrix that consists of all the coefficients of Equation (5), L, $L_{P}$, and $L_{I}$ are the constant items, and W, $W_{P}$, and $W_{I}$ are the weight matrices.

The normal equation with respect to Equation (6) has a well-defined banded and bordered structure [58,59], which is similar to Zheng et al. [57]. Thus, similar to common bundle adjustment, the solution can be achieved by an iterative least squares estimation process (ILSEP). ILSEP is able to make full use of the symmetric and positive, definite and sparse properties of the normal equations and employ the Cholesky method to reduce the computational burden for solving the normal equations [58,59]. Once the correction terms are solved, the exterior orientation parameters, the object point coordinates, and the intrinsic parameters are then corrected accordingly. Those corrected parameters are seen as the initial values for the next iteration until the corrections are small enough.

In contrast to close-range registration, some aerial images are usually captured by metric cameras or calibrated cameras, e.g., data II. In this case, the intrinsic parameters are exactly known, so the correction terms of the intrinsic parameters become unnecessary in the registration. As a result, the weight matrix with respect to the virtual observation equations of the intrinsic parameters can be then set as large as possible to force the correction terms to be as close to zeros as possible. In other word, different weight matrices can be used to adapt to a wide variety of cameras, including metric cameras in traditional airborne photogrammetry and non-metric cameras in low altitude photogrammetry.

Besides, the weight with respect to Equation (5) can be estimated by using equation as follows:

(7) $$w_{P}=w\frac{\sigma_{I}^{2}}{\sigma_{L}^{2}},$$

where w is the weight with respect to the Equation (3), and is set as 1 in the experiments; $\sigma_{I}$ represents the matching precision of the optical images; $\sigma_{L}$ represents the accuracy of the LiDAR data.

As the matching precision of the images and the accuracy of the LiDAR data are unknown for the experiment data, $\sigma_{I}$ and $\sigma_{L}$ are empirically set to 0.5 pixel and 0.5 average point distance of the LiDAR data respectively in this paper, i.e.,:

(8) $$\{\begin{array}{c} \sigma_{I}=0.5\\ \sigma_{L}=\frac{f}{H}\times \frac{\delta }{2}\times \frac{1}{\Delta } \end{array},$$

where f is the focus length, H is the flying height, δ is the average point distance of the LiDAR data, and Δ is the pixel size.

## 3. Implementation

### 3.1. Implementation Flow

**Figure 2:**
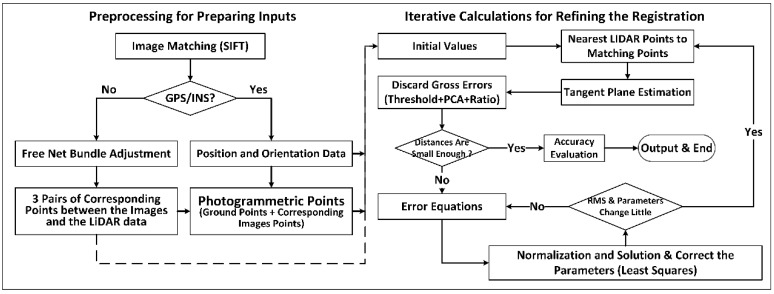
Implementation flow of the registration of aerial optical images with LiDAR data.

The implementation flow of the extended method can be divided into preprocessing for preparing the inputs and iterative calculations for refining the registration, as shown in Figure 2.

Though the extended registration method need not to match the aerial optical images with the LiDAR intensity or depth images, the matching between different optical images are important. The reason is that matching between different optical images provide the redundancy for connecting the LiDAR data with the aerial images. In this paper, scale-invariant feature transform (SIFT) operator [60] is chosen to do the optical image matching, just because SIFT-based matching has the property of being invariant to image scaling, image rotation, and partial illumination change.

In addition, if extra GPS/INS data is available, the initial values of the ground point coordinates can be estimated directly by intersecting the conjugate rays of the aerial optical images. Otherwise, the free net bundle adjustment module of DPGrid [61] is then employed to calculate the exterior orientation parameters of the images and construct the ground point coordinates under an arbitrary coordinate system. Thereafter, similar to Zheng et al. [57], 3 pairs of coarse conjugate points between the free net and the LiDAR data are then selected manually to estimate the initial values of the exterior orientation parameters and the object point coordinates. Such initial values are regarded as the inputs of the iterative calculations, as shown in Figure 2.

In the iterative calculations, the exterior orientation parameters, the intrinsic parameters, and the ground point coordinates are refined simultaneously by solving Equation (6). Such procedure can minimize the distances from the photogrammetric matching points to the corresponding tangent planes of the terrestrial LiDAR data surface. However, the exact tangent plane corresponding to a certain photogrammetric matching point is difficult to identify, so the approximate tangent plane at the closest LiDAR point to the estimated photogrammetric matching point is used in practice. For this reason, the calculations is actually an iterative procedure as follows (Figure 2):

(1) Preprocess the data for estimating the initial values, i.e., the exterior orientation parameters, the object point coordinates, and the intrinsic parameters;
(2) Find the closest 3D point to the photogrammetric matching point from the LiDAR data, and fit a local plane to estimate the normal vector using the surrounding LiDAR points;
(3) Discard the gross 3D points and check if the distances from the photogrammetric matching points to the corresponding tangent planes are all small enough to go to step 8. Otherwise, go to step 4.
(4) Construct the error equations and normal equations with the initial parameters, and then reduce the structure parameters (the corrections of the coordinates of the 3D points) of the normal equations;
(5) Solve the reduced normal equations for acquiring the corrections of the exterior orientation parameters and the intrinsic parameters, and further obtain the corrections of the ground point coordinates with back-substitution;
(6) Correct the parameters and estimate the unit weighted root mean square error (RMSE);
(7) Check if the RMSE or the corrections are small enough to go to step 2. Otherwise, go to step 4, using the corrected parameters as the initial parameters;
(8) Evaluate the accuracy and output the results.

It is obvious that the photogrammetric matching points are much sparser than the LiDAR data. Hence, to reduce the calculation time, instead of calculating all the normal vectors of the LiDAR data during preprocessing, we just estimated the normal vectors of the closest points to the photogrammetric matching points during the iterative calculations.

What is more, the airborne data is different from the terrestrial data, e.g., airborne LiDAR data is generally much greater in size than those of close-range data, and there may be some discontinuities against to the smoothness hypothesis of the LiDAR data surface. The solutions to these problems are further introduced in the following two sections.

### 3.2. Organization Structure of the LiDAR Data

**Figure 3:**
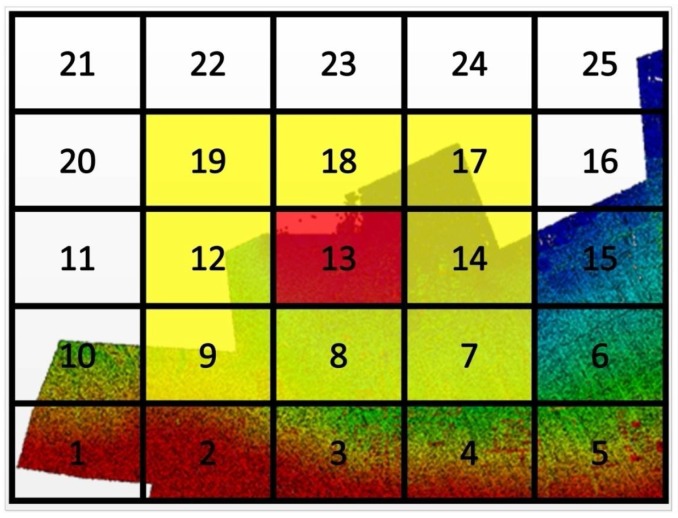
An example of the partition strategy of the LiDAR data.

As mentioned previously, the airborne LiDAR data generally contains so many 3D points that we are difficult to process them. To overcome this difficulty, the LiDAR data is divided into several small blocks in the procedure. Specifically, we partition the LiDAR data into different blocks according to their XY-coordinates in the size of W×H (4 km×4 km for each small block in this paper), as shown in Figure 3.

To further improve the computational efficiency, we make use of a K-D tree [62] to manage the LiDAR points for each small block. K-D tree is known as a space-partitioning data structure for organizing points in a k-dimensional space, and it was useful for us to efficiently search the closest point of a certain photogrammetric matching point from a small LiDAR data block.

Once the LiDAR data is partitioned, the photogrammetric matching points are also classified into those blocks according to their current ground coordinates. Thus, the closest point search in step 2 of the iterative calculations can then be done block by block. As the current ground coordinates of the photogrammetric matching points are coarse in the iterations, the closest point of a certain photogrammetric matching point and its neighborhood may not be exactly located in the classified block, but also possibly in the neighbor blocks. For example, as shown in Figure 3, suppose that a photogrammetric matching point is classified into the 13th block, but its closest LiDAR point and its neighborhood may be located in the 7th, 8th, 9th, 12th, 13th, 14th, 17th, 18th, or 19th blocks in actual. Therefore, to find the closest LiDAR point of the photogrammetric matching point (and the neighborhood of closest LiDAR point), we can make use of the K-D trees to do the search respectively in the 7th, 8th, 9th, 12th, 13th, 14th, 17th, 18th, and 19th blocks at first, and then further find the optimum from those search results.

In addition, the strategy to do the search block by block in order is used in this paper. Take Figure 3 for example, the search are done in order of the shown numbers. If the search of the photogrammetric matching points classified in a block is finished, we can then go to the next neighbor block. In such case, only a part of the current blocks needs to be reloaded and updated, e.g., when we go to the 14th block from the 13th block, only the 9th, 12th, and 19th blocks need to be updated by the 6th, 15th, and 16th blocks respectively. With such strategy, the iterative calculations can be easily applied to large-scale geometric registration of aerial images with LiDAR data with thousands of millions of points.

### 3.3. Discard the Gross Points

There are several inevitable factors that need to be eliminated in the geometric registration procedure. Generally, the main factors that need to be eliminated can be summarized as follows: (1) a few patches of the surface are discontinuities and against to the smoothness hypothesis, e.g., the vegetation regions, and some corners and edges; (2) the ranges of the aerial images and the LiDAR data are not exactly consistent to each other; (3) inevitable gross points are sometimes produced during the image matching.

To eliminate first two factors, when the nearest point to a photogrammetric matching point has been searched, principal component analysis (PCA) is used to estimate the normal vector [63], where the eigenvector with respect to the minimum eigenvalue can be seen as the estimation of the normal vector. The PCA was done by using the 3 × 3  covariance matrix of the 3D coordinates of the first 10 nearest neighbor LiDAR points to the nearest LiDAR point of the photogrammetric matching point. Actually, according to our experience, we can also set the neighborhood size as 10–15 points. Such setting is able to obtain similar estimation results without obviously difference from neighborhood size of 10.

Suppose the distance from the photogrammetric matching point to its nearest point in the LiDAR data is d, and the eigenvalues of the covariance matrix are $\lambda_{1}\geq \lambda_{2}\geq \lambda_{3}\geq 0$.

**Figure 4:**
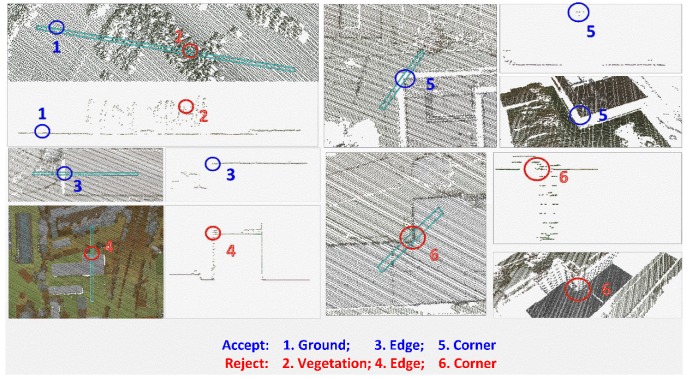
Cases for discarding the gross points.

Given two thresholds, $T_{d}$ for identifying the different ranges, and $T_{\lambda }$ for identifying the discontinuities: if $d\geq T_{d}$, or $\frac{\lambda_{3}}{\lambda_{1}+\lambda_{2}+\lambda_{3}}\geq T_{\lambda }$, the nearest point is then discarded as gross points. Several cases for accepting/rejecting the closest LiDAR points are illustrated in Figure 4.

Note that, the observation number is usually large for the registration, so we only need to discard the gross points that are relative obvious. As a result, $T_{d}$ and $T_{\lambda }$ can empirically set as some conservative values ($T_{d}=2\delta$ and $T_{\lambda }=\frac{1}{6}$ for our experiments).

Though the PCA method is effective to detect most of the significant gross points caused by discontinuities and different ranges, the strategy proposed by Zheng et al. [57] is still needed to eliminate the influence of the gross points caused during the image matching. The strategy is started by giving a gross error rate signed ε (ε=5 for the experiments) followed by the following steps: (1) The photogrammetric matching points are firstly put in order according to the distances to the LiDAR data surface; (2) Then, ε percent of the photogrammetric matching points with the greatest distances are removed as gross points; (3) Finally, the calculations is done by using the remaining points.

If the discontinuities or the range differences is considered, we need just discard the gross nearest LiDAR points rather than the photogrammetric matching points. In this case, Equation (5) are missing, while corresponding collinearity Equation (3) can be remained. The remaining collinearity equations can still provide the connection constraints for the aerial optical images. Therefore, the iterative calculations is also able to adapt to the case that the ranges of the aerial images and the LiDAR data are not well consistent to each other. Besides, an average of at least 20 valid patches per optical image is considered in the experiments. As a result, a minimum of about 20 valid patches per image can be suggested for the registration.

### 3.4. Assess the Registration

As shown in Figure 4, the results are assessed after the iterative calculations. As in any other least squares solution and Zheng et al. [57], the unit weighted root mean square (RMS) error is firstly used to evaluate the iterative calculations. Base on Equation (6), the expression is as follows:

(9) $$RMS_{0}=\sqrt{\frac{V^{T}WV+V_{P}^{T}W_{P}V_{P}+V_{I}^{T}W_{I}V_{I}}{n-n_{T}-n_{P}-n_{I}}},$$

where n is the number of the observations, and $n_{T}$, $n_{P}$, and $n_{I}$ are the numbers of exterior parameters, ground 3D coordinates for the photogrammetric matching points, and intrinsic parameters, respectively. In addition, the unit weighted RMS of the photogrammetric image matching point observations and the distance observations are also used to evaluate the iterative calculations. They can be expressed as follows:

(10) $$RMS_{I}=\sqrt{\frac{V^{T}WV}{n_{Im}}},$$

(11) $$RMS_{d}=\sqrt{\frac{V_{P}^{T}W_{P}V_{P}}{n_{d}}},$$

where $n_{Im}$ and $n_{d}$ are the number of image point observations and the distance observations.

Check point (CP) is another one of the most often used accuracy assessment method in photogrammetry. The main steps can be summarized as follows:

(1) Measure the 3D coordinates corresponds to the CPs from the aerial optical images by using forward intersection, $P_{i}(X_{i},Y_{i},Z_{i})$ (i=1,2,⋯,n, and n is the number of the CPs);
(2) Calculate the errors by comparing the measured $P_{i}$ with the coordinates of the corresponding CP ${\tilde{P}}_{i}({\tilde{X}}_{i},{\tilde{Y}}_{i},{\tilde{Z}}_{i})$,
  (12) $$\Delta_{i}={\tilde{P}}_{i}-P_{i}=(\Delta X_{i},\Delta Y_{i},\Delta Z_{i}),$$
  where:
  (13) $$\left\{ \begin{array}{l} \Delta X_{i}={\tilde{X}}_{i}-X_{i}\\ \Delta Y_{i}={\tilde{Y}}_{i}-Y_{i}\\ \Delta Z_{i}={\tilde{Z}}_{i}-Z_{i} \end{array}\right.,$$
(3) Calculate the statistics of the errors of CPs, for example, the minimum error (MIN), the maximum error (MAX), the mean of the errors ($\bar{\Delta }$), and the root mean square errors (σ),
  (14) $$\bar{\Delta }=({\bar{\Delta }}_{X},{\bar{\Delta }}_{Y},{\bar{\Delta }}_{Z})=(\frac{1}{n}\sum_{i=1}^{n}\Delta X_{i},\frac{1}{n}\sum_{i=1}^{n}\Delta Y_{i},\frac{1}{n}\sum_{i=1}^{n}\Delta Z_{i}),$$
  (15) $$\sigma =(\sigma_{X},\sigma_{Y},\sigma_{Z})=\left(\sqrt{\frac{1}{n}\sum_{i=1}^{n}\Delta X_{i}^{2}},\sqrt{\frac{1}{n}\sum_{i=1}^{n}\Delta Y_{i}^{2}},\sqrt{\frac{1}{n}\sum_{i=1}^{n}\Delta Z_{i}^{2}}\right),$$
  (16) $$\sigma_{XY}=\sqrt{\sigma_{X}^{2}+\sigma_{Y}^{2}},$$

**Figure 5:**
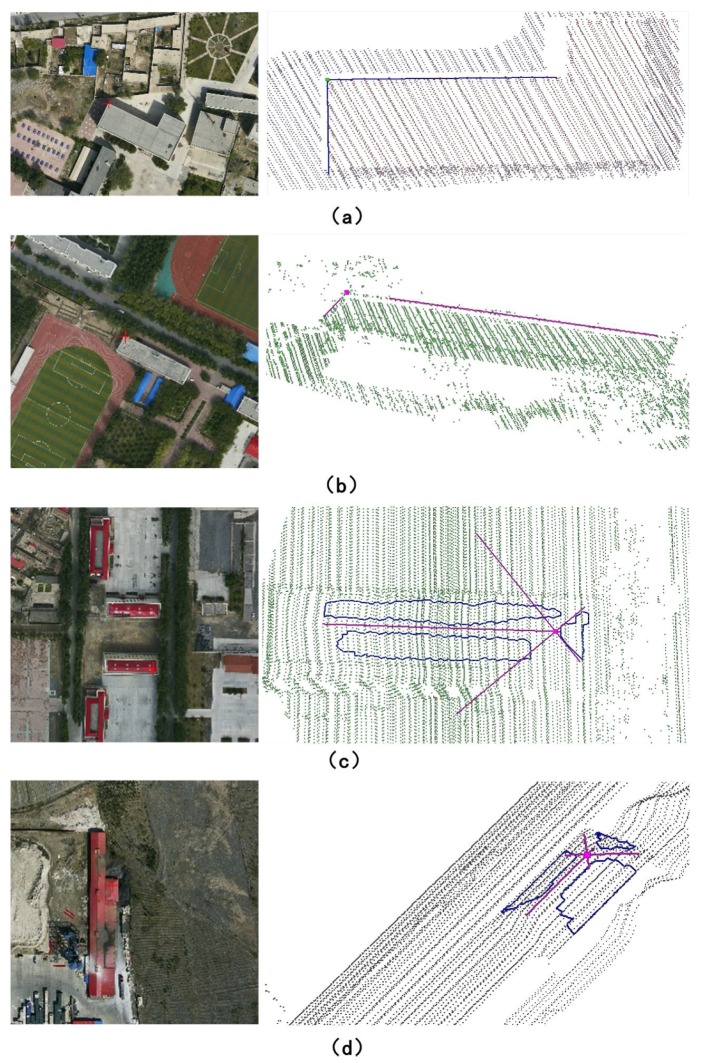
Methods to measure the 3D coordinates of the CPs from LiDAR data: the right is the 3D points measured from the LiDAR data, and the left is the corresponding image points; (**a**,**b**) are measured by using the intersection of two artificial line segments; (**c**,**d**) are measured by using the intersection of three different artificial planes.

Measuring the 3D coordinates ($P_{i}$) from the aerial optical images can be done easily by using photogrammetric software such as DPGrid developed by Wuhan University, so we just need to focus on how to measure the CPs from the LiDAR data. To make the measurement being more accurate, only some artificial feature points are selected as our CPs in this paper. As shown in Figure 5, we need to manually select some LiDAR points along the artificial lines or in the artificial planes at first, and then fit their equations. Accordingly, the 3D coordinates of the CPs (${\tilde{P}}_{i}$) are finally calculated by using the intersections of two artificial line segments or three artificial planes.

## 4. Results

### 4.1. Results of Unit Weighted RMS

The unit weighted RMS of the experiments is estimated by Equations (7)–(9) after the registration, and the results are illustrated in Table 2. The table shows that the unit weighted RMS of the photogrammetric image matching point observations (RMS_I_) is able to reach a sub-pixel level (0.45 to 0.62 pixel), and the unit weighted RMS of the distance observations (RMS_d_) ranges from 0.18 to 0.34 m, i.e., about 0.27 to 0.4 times of the average point distance of the LiDAR data.

### 4.2. Re-Projection of the LiDAR Data

**Figure 6:**
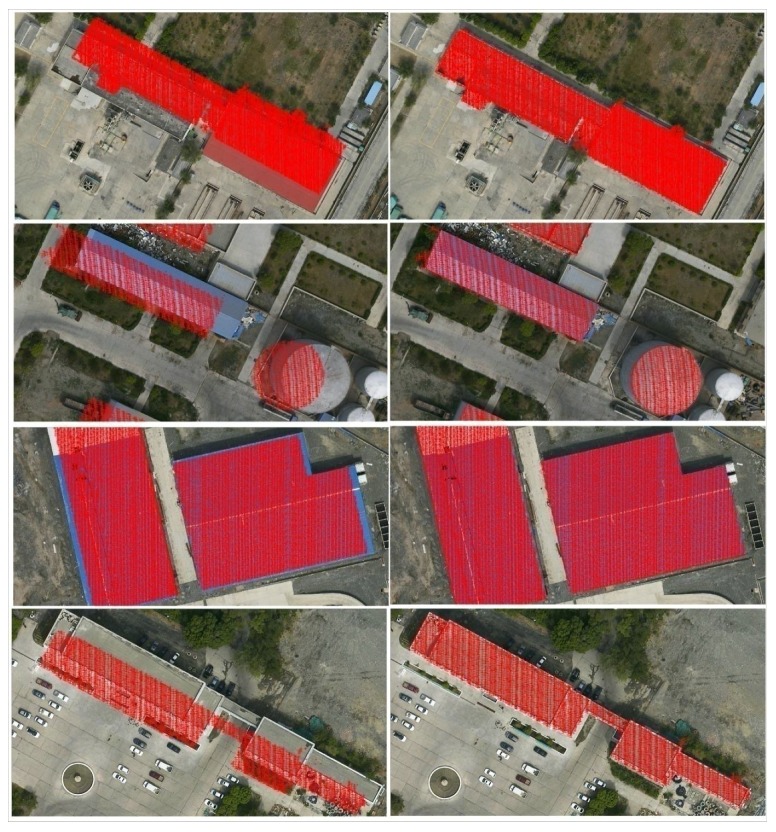
Re-projection of the sub-LiDAR-data to the optical images (Left: before the iterative calculations; Right: after the iterative calculations).

**Figure 7:**
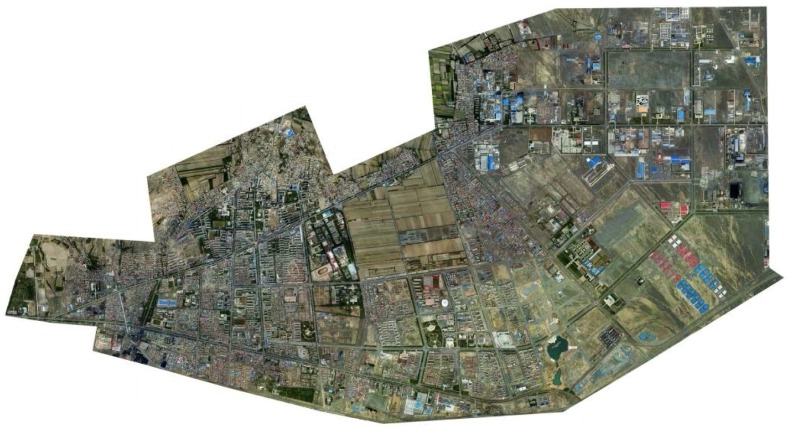
Orthophoto map of data I generated by using the registered LiDAR data and images.

To verify the correctness of the iteration calculations, we further re-project several typical subsets of the LiDAR data to the aerial optical images. As shown in Figure 6, there are obvious biases between the optical images and the re-projection of the LiDAR data before the iterative calculations of the registration, while they are seen to be eliminated by the iterative calculations. Furthermore, take data I as an example, the correctness can also illustrate coarsely from the orthophoto map generated by using the registered LiDAR data and images, as shown in Figure 7.

### 4.3. Statistics of the Check Point Errors

**Figure 8:**
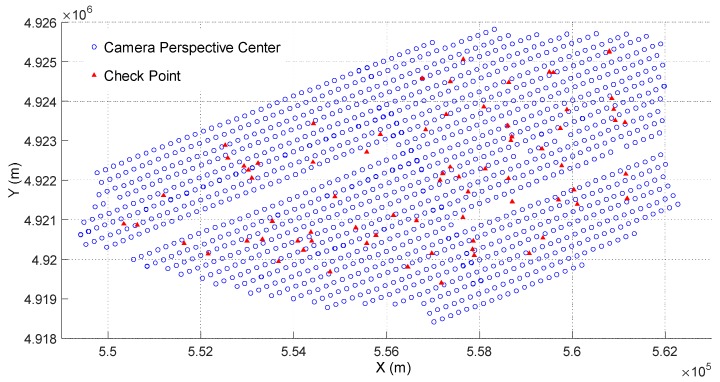
Distribution of the check points and camera perspective centers of data I.

To further test the actual accuracy, several CPs are measured by using the method described in Figure 5. In this paper, the numbers of the CPs of data I–IV are 74, 42, 16, and 14, respectively. Take data I as an example, the distribution of the CPs are laid out widely and evenly in the survey areas, as shown in Figure 8.

**Figure 9:**
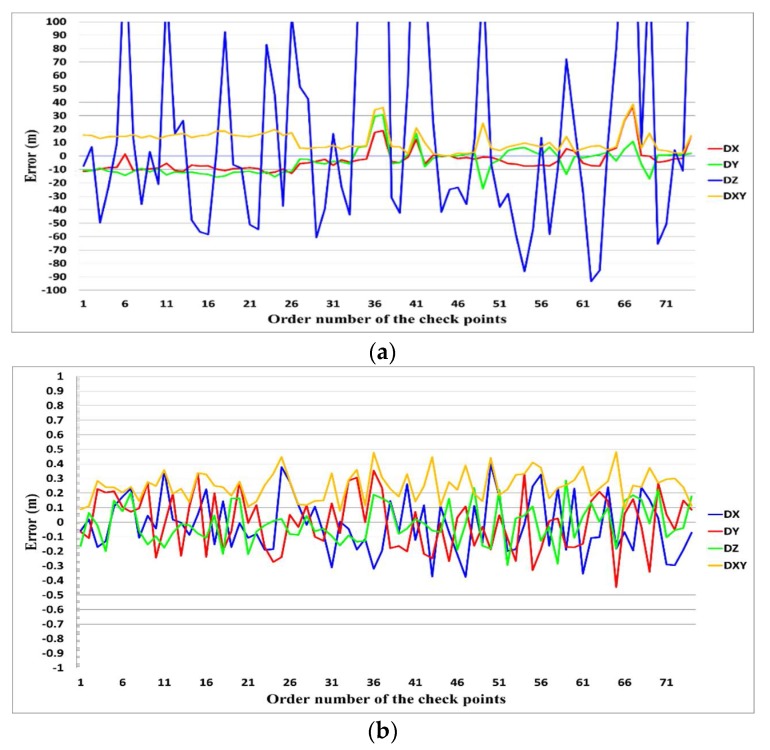
Errors of the check point (data I): (**a**) before the iterative calculations; (**b**) after the iterative calculations.

The actual errors are evaluated by using the differences of the CP coordinates between those measured from the optical images and the LiDAR data, i.e., Equations (10) and (11). Take the case of data I, the errors of the CPs are shown in Figure 9. The figure shows that the accuracy of the registration is greatly improved by the iterative calculations. Such conclusion can be further verified by using the statistics of the errors before and after the iterative calculations, as shown in Table 3. The horizontal accuracy is able to reach 1/4 to 1/2 of the average point distance of the LiDAR data after the iterative calculations, while the vertical accuracy can be improved to 1/8 to 1/4 of the average point distance of the LiDAR data by the iterative calculations.

## 5. Discussion

### 5.1. Discussion on the Accuracy

Since the RMS_I_ ranges from 0.45 to 0.62 pixel, we can firstly conclude that the matching precision of the optical images should reach a sub-pixel level. Furthermore, the RMS_d_ is improved to approximately 0.18 to 0.34 m by the iterative calculations, which is much less than the average point distance of the LiDAR data. As a result, the model and the solution are proved to be feasible for the bundle adjustments in the registration. On the other hand, the results of the re-projection of the LiDAR data can further verify the correctness for the registration, as the biases between the optical images and the re-projection of the LiDAR data can be eliminated by the iterative calculations in the registration, as shown in Figure 6.

Except for the feasibility and the correctness, the actual accuracy is assessed by comparing object point coordinates measured from the registered optical images with the corresponding 3D coordinates measured from the LiDAR data. Both the qualitative visualization (Figure 9) and the quantitative statistics (Table 3) of the check point errors show that the actual accuracy can be greatly improved and reach a relative high level with the iterative calculations. The conclusion can be further enhanced by comparing the actual accuracy with some results of other authors. For example, Table 4 and Table 5 show several methods provided by some other authors together with corresponding error statistics in the past.

Comparing with Table 3, we can find that most of the errors for our experiments are smaller than those of Table 5. The horizontal accuracy ranges from 0.25 m to 1.24 m for those authors, but only 0.17 m to 0.27 m for us. And the vertical accuracy of those authors is about 0.13 m to 1.06 m, while ours is 0.10 m to 0.15 m. Such comparisons still hold even when the error statistics are converted to multiples of the average LiDAR point distance. For those authors, the horizontal accuracy is approximately 0.25 to 1.8 times of the average point distance, while 0.13 to 1.56 times for the vertical accuracy. Besides, as shown in Table 4, the optical image number of those authors are generally much less than our experiments. As a result, we are confident to enhance that the iterative calculation method can reach a relative high level even for large-scale aerial optical images and airborne LiDAR data.

### 5.2. Discussion on the Efficiency

In addition to accuracy, efficiency is also very important for registration in practice. Hence, we further take data I as an example to count the total running time for the registration method in this paper. Our program was developed by using C++ Language and executed with an Asus notebook (Intel (R) Core (TM) i5-2450M CPU@2.50 GHz (4 CPUs), ~2.50 GHz, 4096 MB RAM, Windows 7) in a single thread. The time was counted from the beginning of the preprocessing for preparing the inputs to the end of the iterative calculations for the refinement. The result show that about 31.5 h (1892 min) was consumed for registering the 1432 aerial optical images with the LiDAR data, i.e., an average of 79 s per image.

According to the literatures collocated by us, few authors have reported the time consumption for their registration, except for Kim et al. [36]. The registration of Kim et al. [36] is based on plane estimation and alignment for depth consistency between the LiDAR depth map and the optical image depth map generated by using edge-preserving dense matching. According to their reports, an average of 131 s per 1000 × 688 image is required for the registration. As the frame size of the aerial optical image used in our experiments are much larger than those of Kim et al. [36] (6732 × 8984 and 7680 × 13,824 vs 1000 × 688), we can declare that the time consumption of our method is satisfactory and acceptable in practice. Note that our program is run in a single thread, so if multi-threads or a higher performance computer are used, the efficiency should be further greatly improved.

### 5.3. Discussion on Some Supplementary Notes

When we compare the results of unit weighted RMS with the statistics of the CP errors more carefully, we find that actual horizontal RMS error ($\sigma_{XY}$) is greater than the GSD of the aerial optical images, but the RMS_I_ can reach a sub-pixel level. However, they are actually not conflicting. The RMS_I_ can mainly indicate the matching precision of the aerial optical images, so the RMS_I_ is able to be less than a pixel. Nevertheless, the actual accuracy of the registration is not only decided by the image matching precision, but also the average point distance and the accuracy of the LiDAR data. Though the accuracy is unknown, the average point distance of the LiDAR data is much larger than the GSD for our experiments. This can explain the reason why the RMS_I_ can reach a sub-pixel level while $\sigma_{XY}$ is greater than the GSD.

As shown in Table 3, another unusual thing is that the actual vertical RMS error ($\sigma_{Z}$) is seen to be smaller than $\sigma_{XY}$. In fact, such a phenomenon was also happened in other research, as shown in Table 5. The reason can be explained by using the results provided by Kaartinen et al. [64]: in contrast to traditional photogrammetry, airborne LiDAR data usually has a higher vertical accuracy and a lower horizontal accuracy. For the experiments, the GSD of the aerial optical images is much higher than the average point distance of the LiDAR data, so the actual registration accuracy should be determined mainly by the accuracy of the LiDAR data. In other words, it is similar to the LiDAR data that the actual horizontal accuracy is lower than the vertical.

Note further that our experimental data also has the following characteristics as shown in Table 1: (1) All the data sets mixed urban areas together with rural areas; (2) Data III and IV share the same point clouds, but their aerial optical images are captured in different time; (3) The data contains both the cases with calibrated and uncalibrated optical images. Although the experimental data has so many different characteristics, there is no significant difference being found in registration results for different data sets. So, we can finally declare that the method has broad applicability across the geometric registration of aerial optical images with airborne LiDAR data.

## 6. Conclusions

In this paper, a method to register close-range optical images with terrestrial LiDAR points is extended to large-scale aerial optical images and airborne LiDAR data. The extended method is implemented by minimizing the distances from the photogrammetric matching points to the LiDAR data surface. It can not only keep the advantages of implementation without linear and planar feature extraction and non-rigid deformation correction without extra camera calibration or ground control points, but also prove to have broad applicability across the geometric registration of variety of large-scale aerial optical images with airborne LiDAR data. The experiments show that the actual accuracy can be greatly improved and reach a relative high level with the iterative calculations, i.e., 1/4 to 1/2 of the average point distance of the LiDAR data for the horizontal accuracy, and 1/8 to 1/4 for the vertical accuracy. In addition, the statistics of the running time can further show that the efficiency of the extended method is satisfactory and acceptable in practice. Therefore, the extended method is accurate, feasible, efficient, and practical for broad registration of large-scale aerial optical images and airborne LiDAR data.

Although the method is proved to be practical for the registration of large-scale aerial optical images with airborne LiDAR data in the experiments, there are still a few places left for improvement in our future work. For example, $\sigma_{I}$ and $\sigma_{L}$ are simply set to 0.5 pixel and 0.5 average point distance of the LiDAR data respectively for determining the weight with respect to Equation (5). As a result, the accuracy of the LiDAR data is not really taken into account for the adjustment. Hence, further studies are still needed on the determination of the weight with respect to Equation (5) or the estimation of $\sigma_{I}$ and $\sigma_{L}$. Besides, if the LiDAR data is approximately a whole plane, the calculation becomes ill-conditioned. One possible solution is to combine the method with other point matching or linear feature-based approaches. To further improve the method, we will plan to try our best to solve those problems in the future.

## Figures and Tables

**Table 1:** Information on the aerial optical images and airborne LiDAR data ^1^.

Data		I	II	III	IV
Images	Pixel Size (mm)	0.006	0.006	0.012	0.012
	Frame Size (pixel)	6732 × 8984	6732 × 8984	7680 × 13,824	7680 × 13,824
	Focal Length (mm)	51.0	51.0	120.0	120.0
	Flying Height (m)	900	700	1800	1700
	GSD (m)	0.10	0.09	0.18	0.17
	Forward Overlap	80%	60%	80%	65%
	Side Overlap	75%	30%	35%	20%
	Image Number	1432	222	270	108
	Stripe Number	26	6	8	4
LiDAR Data	Point Distance (m)	0.5	0.5	0.9	
	Point Density (pts/m^2^)	4.0	4.8	1.3	
	Point Number	183,062,176	251,893,187	273,780,202	
	Stripe Number	-	6	12	
	File Number	424	6	12	

^1^ (a) All the data sets mixed urban areas with rural areas, but most artificial control point-based and linear and planar feature-based methods are only available in urban areas; (b) Data III and IV share the same point clouds; (c) the acquire time of the optical images of data IV is different from the LiDAR data, and this was rarely considered in common research; (d) Except for data II, all the cameras of other three data sets are uncalibrated.

**Table 2:** Results of the unit weighted RMS of the registration.

Data	RMS_0_	RMS_I_ (mm)	RMS_d_ (m)
I	0.0022	0.0027	0.18
II	0.0026	0.0037	0.20
III	0.0036	0.0052	0.34
IV	0.0033	0.0062	0.24

**Table 3:** Error statistics before and after the iterative calculations of the registration (Unit: m).

Data		Before the Iterative Calculations					After the Iterative Calculations				
		MIN	MAX	$\bar{\Delta }$	σ	$\sigma_{XY}$	MIN	MAX	$\bar{\Delta }$	σ	$\sigma_{XY}$
Ⅰ	$\Delta_{X}$	−12.86	36.92	−3.175	9.270	13.86	−0.375	0.400	−0.020	0.192	0.270
	$\Delta_{Y}$	−24.38	30.69	−3.982	10.30		−0.445	0.355	−0.004	0.189	
	$\Delta_{Z}$	−93.31	355.9	24.747	97.13		−0.293	0.286	−0.012	0.134	
Ⅱ	$\Delta_{X}$	−0.750	0.964	0.080	0.369	0.437	−0.205	0.282	0.027	0.126	0.165
	$\Delta_{Y}$	−0.489	0.499	0.061	0.235		−0.185	0.199	−0.008	0.107	
	$\Delta_{Z}$	−7.049	1.592	−2.160	3.035		−0.130	0.196	0.031	0.096	
Ⅲ	$\Delta_{X}$	−0.508	0.271	−0.081	0.219	0.585	−0.271	0.241	−0.053	0.159	0.225
	$\Delta_{Y}$	−0.434	1.048	0.389	0.542		−0.225	0.290	0.063	0.158	
	$\Delta_{Z}$	−0.984	1.617	0.237	0.710		−0.147	0.230	0.066	0.150	
Ⅳ	$\Delta_{X}$	−0.299	1.136	0.518	0.644	0.806	−0.161	0.289	0.038	0.147	0.218
	$\Delta_{Y}$	−0.183	0.917	0.393	0.486		−0.276	0.227	−0.040	0.161	
	$\Delta_{Z}$	−1.746	2.207	0.014	0.937		−0.179	0.246	0.024	0.120	

**Table 4:** Information on the data and the methods of some other authors for the registration ^1^.

Author	Image GSD (m)	Image Number	LiDAR Point Distance (m)	CP Number	Method
Kwak et al. [31]	0.25	- ^4^	0.68	13	Bundle adjustment with centroids of plane roof surfaces as control points.
Mitishita et al. [32]	0.15	3	0.70	19	Bundle adjustment with the centroid of a rectangular building roof as a control point.
Zhang et al. [33] ^2^	0.14	8	1.0	9	(1) Bundle adjustment with control points extracted by using image matching between the LiDAR intensity images and the optical images; (2) Bundle adjustment with building corners as control points
Xiong [34]	0.09	84	0.5	109 ^3^	Bundle adjustment with multi-features as control points.

^1^ Xiong [34] is supervised by Zhang [33], so the method proposed by Xiong [34] can be seen as a development of the methods provided by Zhang et al. [33]; ^2^ Zhang et al. [33] provided both the results of bundle adjustment with building corners and bundle adjustment with matching points; ^3^ 37 horizontal CPs and 72 vertical CPs; ^4^ Kwak et al. [31] didn’t provided the image number of their experiments.

**Table 5:** Error statistics provided by authors with respect to Table 4 (Unit: m).

Author	$\sigma_{X}$	$\sigma_{Y}$	$\sigma_{XY}$	$\sigma_{Z}$
Kwak et al. [31]	0.76	0.98	1.24	1.06
Mitishita et al. [32]	0.21	0.31	0.37	0.36
Zhang et al. [33] ^1^	0.24	0.28	0.37	0.23
Zhang et al. [33] ^2^	0.16	0.19	0.25	0.13
Xiong [34]	0.23	0.22	0.33	0.13

^1^ Accuracy of the registration implemented by using bundle adjustment with control points extracted by image matching between LiDAR intensity images and optical images; ^2^ Accuracy of the registration implemented by using bundle adjustment with building corners as control points.
